# Characterizing hospital workers' willingness to report to duty in an influenza pandemic through threat- and efficacy-based assessment

**DOI:** 10.1186/1471-2458-10-436

**Published:** 2010-07-26

**Authors:** Ran D Balicer, Daniel J Barnett, Carol B Thompson, Edbert B Hsu, Christina L Catlett, Christopher M Watson, Natalie L Semon, Howard S Gwon, Jonathan M Links

**Affiliations:** 1 Department of Epidemiology, Faculty of Health Sciences, Ben-Gurion University of the Negev, Beer-Sheva, Israel; 2 Johns Hopkins Preparedness and Emergency Response Research Center, Baltimore, Maryland, USA; 3 Department of Environmental Health Sciences, Johns Hopkins Bloomberg School of Public Health, Baltimore, Maryland, USA; 4 Johns Hopkins Center for Public Health Preparedness, Baltimore, Maryland, USA; 5 Department of Biostatistics, Johns Hopkins Bloomberg School of Public Health, Baltimore, Maryland, USA; 6 Department of Emergency Medicine, Johns Hopkins University School of Medicine, Baltimore, Maryland, USA; 7 Johns Hopkins Office of Critical Event Preparedness and Response, Baltimore, Maryland, USA; 8 Department of Anesthesiology and Critical Care Medicine, Johns Hopkins University School of Medicine, Baltimore, Maryland, USA; 9 Department of Pediatrics, National Naval Medical Center, Bethesda, Maryland, USA; 10 Office of Emergency Management, Johns Hopkins Hospital, Baltimore, Maryland, USA

## Abstract

**Background:**

Hospital-based providers' willingness to report to work during an influenza pandemic is a critical yet under-studied phenomenon. Witte's Extended Parallel Process Model (EPPM) has been shown to be useful for understanding adaptive behavior of public health workers to an unknown risk, and thus offers a framework for examining scenario-specific willingness to respond among hospital staff.

**Methods:**

We administered an anonymous online EPPM-based survey about attitudes/beliefs toward emergency response, to all 18,612 employees of the Johns Hopkins Hospital from January to March 2009. Surveys were completed by 3426 employees (18.4%), approximately one third of whom were health professionals.

**Results:**

Demographic and professional distribution of respondents was similar to all hospital staff. Overall, more than one-in-four (28%) hospital workers indicated they were not willing to respond to an influenza pandemic scenario if asked but not required to do so. Only an additional 10% were willing if required. One-third (32%) of participants reported they would be unwilling to respond in the event of a more severe pandemic influenza scenario. These response rates were consistent across different departments, and were one-third lower among nurses as compared with physicians. Respondents who were hesitant to agree to work additional hours when required were 17 times less likely to respond during a pandemic if asked. Sixty percent of the workers perceived their peers as likely to report to work in such an emergency, and were ten times more likely than others to do so themselves. Hospital employees with a perception of high efficacy had 5.8 times higher declared rates of willingness to respond to an influenza pandemic.

**Conclusions:**

Significant gaps exist in hospital workers' willingness to respond, and the EPPM is a useful framework to assess these gaps. Several attitudinal indicators can help to identify hospital employees unlikely to respond. The findings point to certain hospital-based communication and training strategies to boost employees' response willingness, including promoting pre-event plans for home-based dependents; ensuring adequate supplies of personal protective equipment, vaccines and antiviral drugs for all hospital employees; and establishing a subjective norm of awareness and preparedness.

## Background

In the face of well-documented surge capacity limitations, the willingness of hospital-based providers to report to work during a disaster has received increasing attention as a salient policy and planning issue for the public health emergency preparedness system. Pandemic influenza, whether relatively mild or severe, can be expected to strain already-limited hospital resources [1]. This was documented for Pandemic (H1N1) 2009 in some Southern hemisphere countries during the recent winter, where the pandemic presented a variety of surge capacity resource challenges [2,3]. In the United States (US), where influenza cases surged in early fall 2009, the Centers for Disease Control and Prevention predicted that 15 states could run out of available hospital beds during the peak of the outbreak [4].

In such an "all hands on deck" situation, worker absenteeism can be expected not solely due to illness among employees and their families, but also due to voluntary absenteeism. Indeed, a growing body of research points to response willingness rates that are far from universal, with the extent of these willingness gaps varying across different healthcare workforce cohorts, countries, and scenario contexts [5-7]. With regard to hospital workers' views toward pandemic influenza response, for example, a 2006 survey conducted among employees at a Level II trauma center revealed that 42% of respondents answered "maybe" and 8% answered "no" to a question on willingness to respond to this threat [8]. These ambivalent or negative responses suggest that hospital workforce absenteeism may be due, in substantial measure, to attitudinal and related perceptual factors apart from direct illness. Such findings also point toward the need for enhanced understanding of response willingness among other responder cohorts whose failure to report to work (for reasons other than illness), could further compromise the surge capacity of an already-strained healthcare system [9].

We have recently found that Witte's Extended Parallel Process Model (EPPM) [10] -- a behavioral model based on decades of fear appeal research -- can offer a useful lens for understanding how healthcare providers' perceptions of threat and efficacy may positively or negatively influence their willingness to fulfill response expectations [11].

Despite an overall acceleration of survey-based research on willingness to respond in recent years, studies focusing on hospital providers' willingness to report to work in an influenza pandemic have not followed clear theoretical frameworks to analyze the key causes for this behavior [5,7,12-16]. To date, no published study has used the EPPM to examine hospital workers' willingness to respond to any disaster category.

We therefore aimed to identify the relative influences of perceived threat and efficacy on response willingness to pandemic influenza among employees at Johns Hopkins Hospital (JHH), a 984-bed, tertiary-care, academic teaching hospital and Level I trauma center in Baltimore, Maryland, and to uncover additional relevant barriers and facilitators to pandemic influenza response willingness among this healthcare cohort.

## Methods

Research ethics approval for the survey and its administration was received from The Johns Hopkins Medicine Institutional Review Board (JHM IRB) with a waiver of written consent. The JHM IRB-approved study materials included an electronic disclosure describing the study and emphasizing voluntary participation; verbal consent was not requested or required by JHM IRB for this approved study. The survey tool, entitled "Disaster preparedness and emergency response survey", was an anonymous online instrument (SurveyMonkey.com, Portland, OR) consisting of two main sections: a demographic section and an attitude/belief section that focused on hospital workers' attitudes and beliefs toward emergency response. The demographic and professional information included gender, age, hospital affiliation, years of employment in the hospital, hours spent per week working at the hospital, whether the hospital is the primary place of employment, primary departmental affiliation in the hospital, primary role in the department, years of employment in primary departmental role, highest educational level, reliance on public transportation, whether elderly dependents live with the respondent or nearby, whether children live at home, if the respondent is a single parent, and whether they have pets relying directly on their care. These key questions are listed in Table 1.

**Table 1:** Associations between respondents' demographic characteristics and self-reported willingness to respond (WTR) to a pandemic flu emergency

			WTR if required			WTR if asked, but not required		
		**%**^**a**^	**% Agree**^**b**^	**OR**^**c**^	(95%CI)	% Agree	OR	(95%CI)
**All**^**d**^			82.5			72.0		
**By respondent characteristics**								
Gender	Female	73	81.6	Reference		69.9	Reference	
	Male	27	84.9	1.27	(1.00 - 1.61)	77.1	1.45	(1.18 - 1.78)
Age (years)	<30	17	80.6	Reference		66.5	Reference	
	30-39	22	79.8	0.95	(0.68 - 1.32)	65.8	0.97	(0.73 - 1.28)
	40-49	26	82.2	1.11	(0.80 - 1.55)	72.7	1.34	(1.02 - 1.77)
	50-59	27	85.1	1.38	(0.99 - 1.92)	76.3	1.63	(1.23 - 2.14)
	60+	9	84.3	1.29	(0.84 - 1.99)	79.0	1.90	(1.30 - 2.76)
Duration at JHH^e ^(years)	<1	11	81.3	Reference		69.3	Reference	
	1-5	33	82.8	1.10	(0.77 - 1.58)	72.3	1.16	(0.85 - 1.57)
	6-10	17	80.1	0.93	(0.63 - 1.37)	70.0	1.04	(0.74 - 1.45)
	>10	39	83.5	1.16	(0.81 - 1.65)	73.1	1.21	(0.90 - 1.63)
Hours/week working at JHH	<10	4	80.4	Reference		70.9	Reference	
	10-19	1	85.3	1.41	(0.49 - 4.12)	78.8	1.53	(0.60 - 3.90)
	20-29	3	82.7	1.16	(0.54 - 2.52)	64.5	0.75	(0.40 - 1.41)
	30-39	10	79.9	0.97	(0.54 - 1.74)	67.2	0.84	(0.51 - 1.40)
	40-49	68	80.3	0.99	(0.60 - 1.64)	70.1	0.96	(0.62 - 1.50)
	50+	16	92.3	2.93	(1.60 - 5.34)	82.3	1.91	(1.17 - 3.11)
Worked in JHH role (years)	<1	13	81.5	Reference		68.3	Reference	
	1-5	37	82.9	1.11	(0.79 - 1.55)	73.2	1.27	(0.95 - 1.68)
	6-10	17	81.0	0.97	(0.66 - 1.41)	71.1	1.14	(0.83 - 1.58)
	>10	34	83.5	1.15	(0.82 - 1.61)	72.5	1.23	(0.92 - 1.63)
Highest education level completed	Professional	19	90.6	Reference		79.0	Reference	
	MS	20	85.3	0.60	(0.41 - 0.88)	74.5	0.78	(0.58 - 1.04)
	Bachelors	36	82.7	0.50	(0.35 - 0.70)	70.3	0.63	(0.49 - 0.81)
	HS/GECD	24	71.9	0.27	(0.19 - 0.38)	65.5	0.51	(0.38 - 0.67)
Rely on public transportation	No	82	82.9	Reference		72.0	Reference	
	Yes	18	80.1	0.83	(0.63 - 1.09)	71.8	0.99	(0.78 - 1.26)
Have elder family members who rely on you for care	No	78	82.6	Reference		72.5	Reference	
	Yes	22	82.3	0.98	(0.76 - 1.25)	69.8	0.88	(0.71 - 1.08)
Children/marital status	No children	54	83.3	Reference		74.5	Reference	
	Children/single parent	10	80.8	0.81	(0.57 - 1.16)	67.1	0.70	(0.52 - 0.95)
	Children/Married	36	82.0	0.91	(0.73 - 1.14)	69.9	0.80	(0.66 - 0.96)
Have pets who rely solely on you	No	44	83.2	Reference		73.4	Reference	
	Yes	56	81.8	0.91	(0.74 - 1.12)	70.5	0.87	(0.73 - 1.03)
Type of profession	MD	14	90.4	Reference		79.3	Reference	
	RN	17	86.1	0.66	(0.43 - 1.00)	70.2	0.61	(0.45 - 0.84)
	Other professional	3	89.5	0.90	(0.40 - 2.01)	81.6	1.15	(0.62 - 2.16)
	Other (non-professional)	66	78.9	0.40	(0.28 - 0.56)	70.0	0.61	(0.47 - 0.79)
Department type	Emergency medicine	4	85.5	Reference		79.1	Reference	
	Clinical	72	83.5	0.86	(0.50 - 1.49)	71.6	0.67	(0.42 - 1.07)
	Non-clinical	24	79.2	0.65	(0.37 - 1.14)	71.5	0.67	(0.41 - 1.09)

^a ^Percent of respondents in category within characteristic

^b ^Percent agreeing with WTR statement (positive response)

^c ^OR is the odds ratio provided in the logistic regression which compares the odds between a positive WTR response and a negative WTR response with respect to a particular characteristic category compared to its reference category, unadjusted for other demographic characteristics.

^d ^Percent covers all respondents.

^e ^Johns Hopkins Hospital (JHH)

For the pandemic influenza emergency scenario, a series of attitudes and belief statements were presented for level of agreement along with two open-ended questions. Responses to the attitude and belief statements were based on a 9-point Likert scale with a response of '1' indicating strong agreement with the statement, a response of '5' indicating neutrality, and a response of '9' indicating strong disagreement with the statement. Respondents could also indicate "don't know". Two main contexts for willingness to respond ("WTR") to an influenza pandemic [WTR if asked but not required to respond (hereafter referred to as "WTR if asked"), and WTR if required to respond] and several conditional WTR contexts were also presented using the 9-point Likert scale

The survey's EPPM-based threat and efficacy measures have been widely validated by numerous studies in multiple countries, cultural settings, and health contexts [17]. The other statements in the online survey were derived from the Johns Hopkins~Public Health Infrastructure Response Survey Tool (JH~PHIRST) [11]. This survey was based on validated risk communication theory [18,19] in the context of an identified group of likely peripheral risk perception modifiers taken from public health emergency preparedness training experiences [20]. These questions are listed in Table 2. One question in this survey that was not a part of the previous JH~PHIRST survey inquired about workers' willingness to perform their duties during an emergency if more hours were required.

**Table 2:** Associations between attitudes/beliefs and self-reported willingness to respond (WTR) to a pandemic flu emergency

		WTR if required	WTR if asked, but not required
	**% Agree**^**a**^	**OR (95%CI)**^**b**^	OR (95%CI)
**Attitudes and belief statements**			
Perceived likelihood of occurrence in this region	48.9	1.60	1.25
		(1.27 - 2.00)	(1.03 - 1.51)
Perceived severe health consequences likely	83.0	3.06	1.90
		(2.37 - 3.96)	(1.50 - 2.40)
Perceived likelihood of being asked to report to duty	65.5	4.57	2.98
		(3.59 - 5.82)	(2.44 - 3.64)
Perceived likelihood that colleagues will report	62.1	9.41	8.48
		(7.09 - 12.49)	(6.79 - 10.60)
Perceived knowledge about the public health impact	63.8	3.00	2.30
		(2.39 - 3.78)	(1.89 - 2.78)
Perceived awareness of role-specific responsibilities	42.4	2.64	2.22
		(2.01 - 3.45)	(1.80 - 2.75)
Perceived skills for role-specific responsibilities	63.1	5.33	3.50
		(4.14 - 6.86)	(2.86 - 4.28)
Psychologically prepared	66.7	9.51	5.95
		(7.25 - 12.48)	(4.83 - 7.34)
Perceived ability to safely get to work	72.2	10.62	6.72
		(8.17 - 13.80)	(5.43 - 8.32)
Confidence in personal safety at work	56.7	7.44	6.54
		(5.62 - 9.84)	(5.25 - 8.15)
Perceived ability to perform duties (Self Efficacy)	73.4	12.50	7.97
		(9.59 - 16.28)	(6.40 - 9.92)
Perceived that family is prepared to function in absence	59.2	8.64	5.41
		(6.53 - 11.43)	(4.40 - 6.66)
Self-reported willingness to perform duties if additional hours are required	75.5	17.46	13.64
		(13.23 - 23.04)	(10.79 - 17.25)
Hospital's perceived ability to provide timely information	72.6	5.29	4.41
		(4.15 - 6.75)	(3.58 - 5.45)
Perceived ability to address public questions	53.6	4.84	3.88
		(3.70 - 6.33)	(3.15 - 4.78)
Perceived importance of one's role in the agency's overall response	55.2	4.46	3.06
		(3.45 - 5.76)	(2.50 - 3.73)
Perceived need for pre-event preparation and training	88.3	5.44	3.89
		(4.09 - 7.24)	(2.96 - 5.12)
Perceived need for during/post-event psychological support	59.0	1.66	1.32
		(1.33 - 2.08)	(1.09 - 1.60)
Perceived high impact of one's response (Response Efficacy)	72.5	5.89	3.64
		(4.61 - 7.52)	(2.96 - 4.48)

^a ^Percent agreeing with WTR statement (positive response)

^b ^OR is the odds ratio provided in the logistic regression which compares the odds between a positive WTR response and a negative WTR response with respect to the positive statement response compared to the negative statement response, adjusted for key demographic characteristics: gender, age, hours/week worked, highest education level completed and children/marital status

### Study participants

All employees of the Johns Hopkins Hospital (N = 18,612) were designated as eligible for participation in the survey, which was conducted from January 2, 2009 to March 9, 2009 (prior to the current H1N1 pandemic outbreak) in all departments. Study notification and requests for voluntary participation were distributed via department manager announcements, hospital-wide emails, posters, and informational plasma screens throughout the hospital. The importance of participation across all departments and job duties was strongly encouraged. The study was approved by the JHM-IRB.

### Extended Parallel Process Model Methodology

According to the EPPM, in order to be effective, messages must contain two parts: threat and efficacy. According to this model, the threat and efficacy components are processed in parallel by the message recipient, and both components must be accepted by the recipient to achieve the desired behavior or practice (at both individual and collective levels). If the threat portion is not accepted, the message is rejected. If the threat portion is accepted, but the efficacy portion is not, the acceptance of the threat portion alone triggers fear, which can result in maladaptive responses such as denial or avoidance.

In accordance with the methodology validated in previous work [11,20], four scenario-specific profiles for the EPPM were created, based on levels of perceived threat and perceived efficacy. These profiles include: low threat and low efficacy (LT/LE), low threat and high efficacy (LT/HE), high threat and low efficacy (HT/LE), and high threat and high efficacy (HT/HE). Using the Likert-scale responses, the 'threat' variable was determined as the product of the participant's response to the perceived likelihood of the occurrence of the given public health threat and the perceived severity of the event statements, while the 'efficacy' variable was calculated as the product of the participant's response to the statements regarding their perceived ability to perform their duty (Self Efficacy) and their perceived impact on combating the given public health threat (Response Efficacy). Low and high categories of perceived threat and efficacy were determined by the median value of each product, respectively.

### Statistical Analysis

Prior to analysis, responses to the attitude and belief statements were dichotomized into categories of ≤4 ('positive response') versus >5 ('negative response'). One of the four EPPM profiles was assigned to each respondent using the low and high perceived threat and efficacy categories calculated as described above and in previous EPPM survey-based research [11].

Distributions of demographic/professional factors and agreement with attitude/belief statements were obtained with respect to the two main WTR contexts noted above. Univariate logistic regression analyses were performed to determine key demographic factors most predictive of a positive response to these WTR contexts. Multivariate logistic regression analyses, adjusting for the key demographic factors, were then performed to evaluate the attitude/belief statements and EPPM profiles predictive of a positive response for each of the main WTR contexts, and to evaluate the association between demographic characteristics and EPPM profiles. McNemar's test of correlated proportions compared agreement between WTR contexts. Missing and "don't know" responses were excluded from the analyses. All analyses were performed using STATA version 10.1 (STATA Corporation, 2009. College Station, TX).

## Results

Responses to the online survey were received from 3426 respondents whose primary affiliation was with JHH (18.4% response rate). Key characteristics of the respondents are detailed in Table 1. Among the respondents, 27.3% were male, and 72.7% were female; 16.5% were younger than 30 years, 47.5% were aged 30-49 years, and 36% were aged 50 and older. Thirty-four percent of the respondents were clinical staff, and 66% were non-clinical (the latter including food service/linens, IT, legal, executive officers, nursing administration, parking, pharmacy, safety, social workers, supply chain, telecommunications, etc). Of the 1170 clinical respondents, 42.7% were physicians, 49.2% were nurses, and 8.1% were "other" (physician extenders and medical/nursing students). A majority of respondents (60.7%) were working in the hospital for up to 10 years and 39.3% for over 10 years.

As compared with the distribution of survey respondents key characteristics shown in table 1, the entire JHH staff had similar proportional distribution, with 68.2% Females (compared with 72.7%), 11.7% MDs and 15.3% RNs (compared with 14.6% and 16.8%), and 49.8% below the age of 40 (compared with 38.3%). This indicates good sample representativeness, with slight overrepresentation of younger staff.

Willingness to respond to an influenza pandemic scenario was 72% if asked, and 82.5% if required to respond. Table 1 shows the strength of unadjusted associations with WTR if asked (OR(95%CI) for older respondents [OR(95%CI) of 1.34 (1.12, 1.77), 1.63 (1.23, 2.14), and 1.9 (1.30, 2.76) for ages 40-49, 50-59 and 60+, respectively, as compared with ages <30]; and those working 50+ hours per week [OR(95%CI): 1.91 (1.17, 3.11) compared with those working <10 hours per week]. In addition, a significantly lower unadjusted likelihood of WTR if asked was evident for those having children and married [OR(95%CI): 0.8 (0.65, 0.96)] and single parents [OR(95%CI): 0.7 (0.52, 0.95)], compared to those having no children regardless of marital status; a lower level of education [OR(95%CI) of 0.51 (0.38, 0.67)] for HS/GED, and [OR(95%CI) of 0.63 (0.49, 0.81)] for Bachelors, compared to a professional degree; and for nurses and "other" (non-professionals) [OR(95%CI): 0.61 (0.45, 0.84) and 0.61 (0.47, 0.79), respectively] compared to physicians. Other variables, including type of department (emergency medicine, clinical and non-clinical) had no significant association with WTR if asked.

In a multivariate analysis, five of these demographic and professional factors (age, working hours, marital status, dependent children, and level of education) were found to be independently associated with both WTR if asked and WTR if required, and are used as adjustors in subsequent analyses.

After adjusting for these demographic factors, several attitude/belief statements had a significant association with WTR if asked (Table 2): belief that pandemic is likely and of its severe consequences [OR(95%CI): 1.25 (1.03, 1.51) and 1.9 (1.50, 2.40), respectively]; level of perceived knowledge regarding pandemic events [OR(95%CI): 2.3 (1.89, 2.78)]; perceived importance of one's role in the hospital's overall response [OR(95%CI): 3.06 (2.50, 3.73)]; feeling psychologically prepared to perform one's role-specific responsibilities in the event [OR(95%CI): 5.95 (4.83, 7.34)]; perceived confidence one would be safe at work [OR(95%CI): 6.54 (5.25, 8.50)]; perceived confidence one could safely get to work [OR(95%CI): 6.72 (5.43, 8.32)]; perceived ability to perform one's duties [OR(95%CI): 7.97(6.40, 9.92)]; perception of colleagues being willing to report [OR(95%CI): 8.48 (6.79, 10.60)]; perception that family is prepared to function in one's absence [OR(95%CI): 5.41 (4.40, 6.66)]; and willingness to perform their duties if more hours are required [OR(95%CI): 13.64 (10.79, 17.25)].

In accordance with the EPPM, measures for threat and efficacy perception were calculated. When adjusting for the key demographic factors, higher perceived threat [OR(95%CI): 1.23 (1.02, 1.49)] and higher perceived efficacy [OR(95%CI): 5.86 (4.68, 7.41)] were associated with a higher WTR if asked (Table 3). When the threat and efficacy factors were combined into the four EPPM profiles, the high-threat/high-efficacy profile, which comprised 27.3% of all respondents with an EPPM threat/efficacy assignment, was associated with almost nine times higher odds of WTR if required, and over five times higher odds of WTR if asked, as compared to the odds for the reference low-threat/low-efficacy profile [OR(95%CI): 9.25 (5.94, 14.40), and 5.52 (4.03, 7.56), respectively].

**Table 3:** Associations between EPPM^a ^categories and self-reported willingness to respond (WTR) to a pandemic flu emergency

			WTR if required		WTR if asked, but not required	
		**%**^**b**^	**% Agree**^**c**^	**OR (95%CI)**^**d**^	% agree	OR (95%CI)
**By EPPM categories**						
**EPPM - Threat**	Low	51.2	79.0	Reference	69.9	Reference
	High	48.8	86.8	1.58	75.2	1.23
				(1.25 - 1.98)		(1.02 - 1.49)
**EPPM - Efficacy**	Low	51.4	72.6	Reference	58.6	Reference
	High	48.6	95.8	9.33	88.9	5.86
				(6.66 - 13.08)		(4.63 - 7.41)
**EPPM - Combined**	Low Threat/Low Efficacy	30.3	69.3	Reference	57.7	Reference
	Low Threat/High Efficacy	21.2	96.3	13.09	90.1	7.12
				(7.67 - 22.34)		(4.94 - 10.25)
	High Threat/Low Efficacy	21.2	77.6	1.41	60.9	1.10
				(1.05 - 1.90)		(0.85 - 1.42)
	High Threat/High Efficacy	27.3	95.6	9.25	88.6	5.52
				(5.94 - 14.40)		(4.03 - 7.56)

^a ^Extended Parallel Process Model

^b ^Percent of respondents included in category

^c ^Percent agreeing with WTR statement (positive response)

^d ^OR is the odds ratio provided in the logistic regression which compares the odds between a positive WTR response and a negative WTR response with respect to this EPPM category compared to its Reference category, adjusted for key demographic characteristics: gender, age, hours/week worked, highest education level completed and children/marital status.

High efficacy profiles, most significantly the high-threat/high-efficacy profile, had at least some association (Table 4) with age, hours working/week, being responsible for elder family members, children/marital status, and profession type, compared to the low threat/low efficacy profile.

**Table 4:** Associations between EPPM^a ^categories related to a pandemic flu emergency and respondents' demographic characteristics

		High Threat/Low Efficacy		Low Threat/High Efficacy		High Threat/High Efficacy	
	**Overall %**^**b**^	21.20%		21.20%		27.30%	
**By Respondent characteristics**		**MOR**^**b**^	**(95%CI)**	**MOR**	**(95%CI)**	**MOR**	**(95%CI)**
Gender	Female	Reference		Reference		Reference	
	Male	1.33	(1.00 - 1.77)	0.91	(0.69 - 1.22)	1.05	(0.80 - 1.39)
Age (years)	<30	Reference		Reference		Reference	
	30-39	1.15	(0.76 - 1.74)	0.93	(0.62 - 1.39)	1.68	(1.11 - 2.56)
	40-49	1.32	(0.87 - 2.02)	1.05	(0.69 - 1.60)	2.07	(1.36 - 3.15)
	50-59	1.29	(0.87 - 1.90)	0.87	(0.59 - 1.29)	2.21	(1.49 - 3.28)
	60+	1.41	(0.82 - 2.43)	1.54	(0.92 - 2.59)	3.17	(1.89 - 5.30)
Duration at JHH^c ^(years)	<1	Reference		Reference		Reference	
	1-5	1.05	(0.67 - 1.64)	0.77	(0.49 - 1.20)	0.94	(0.61 - 1.45)
	6-10	1.11	(0.67 - 1.86)	0.96	(0.57 - 1.62)	0.95	(0.58 - 1.54)
	>10	1.19	(0.72 - 1.96)	1.28	(0.78 - 2.11)	1.01	(0.63 - 1.61)
Hours/week working at JHH	<10	Reference		Reference		Reference	
	10-19	1.31	(0.35 - 4.84)	0.85	(0.25 - 2.83)	2.41	(0.72 - 8.03)
	20-29	1.39	(0.51 - 3.78)	1.22	(0.50 - 2.96)	1.55	(0.57 - 4.19)
	30-39	0.81	(0.36 - 1.82)	0.72	(0.36 - 1.44)	1.09	(0.49 - 2.41)
	40-49	1.07	(0.52 - 2.20)	0.65	(0.35 - 1.20)	1.36	(0.67 - 2.79)
	50+	1.24	(0.59 - 2.62)	0.74	(0.39 - 1.41)	2.85	(1.35 - 6.01)
Worked in JHH role (years)	<1	Reference		Reference		Reference	
	1-5	1.12	(0.74 - 1.70)	0.91	(0.60 - 1.37)	0.93	(0.63 - 1.38)
	6-10	1.27	(0.78 - 2.08)	1.16	(0.72 - 1.89)	1.08	(0.68 - 1.71)
	>10	1.38	(0.86 - 2.21)	1.38	(0.86 - 2.20)	1.28	(0.82 - 2.00)
Highest education level completed	Professional	Reference		Reference		Reference	
	MS	1.38	(0.89 - 2.14)	0.89	(0.60 - 1.33)	1.43	(0.95 - 2.15)
	Bachelors	1.42	(0.93 - 2.17)	0.71	(0.48 - 1.03)	1.39	(0.94 - 2.06)
	HS/GECD	1.77	(1.12 - 2.80)	0.61	(0.39 - 0.95)	1.19	(0.77 - 1.84)
Rely on public transportation	No	Reference		Reference		Reference	
	Yes	0.96	(0.69 - 1.35)	1.05	(0.74 - 1.48)	0.91	(0.66 - 1.26)
Have elder family members who rely on you for care	No	Reference		Reference		Reference	
	Yes	1.14	(0.83 - 1.56)	1.11	(0.81 - 1.52)	1.44	(1.07 - 1.92)
Children/marital status	No children	Reference		Reference		Reference	
	Children/single parent	1.13	(0.71 - 1.81)	0.81	(0.47 - 1.40)	1.67	(1.09 - 2.56)
	Children/Married	1.10	(0.83 - 1.47)	1.42	(1.06 - 1.89)	1.10	(0.84 - 1.44)
Have pets who rely solely on you	No	Reference		Reference		Reference	
	Yes	0.88	(0.68 - 1.13)	1.21	(0.94 - 1.56)	1.11	(0.88 - 1.40)
Type of profession	MD	Reference		Reference		Reference	
	RN	1.90	(0.94 - 3.84)	0.84	(0.44 - 1.61)	2.77	(1.50 - 5.13)
	Other professional	3.53	(1.32 - 9.44)	1.78	(0.71 - 4.49)	3.72	(1.51 - 9.17)
	Other (non-professional)	1.18	(0.64 - 2.19)	0.82	(0.48 - 1.40)	1.21	(0.71 - 2.07)
Department type	Emergency medicine	Reference		Reference		Reference	
	Clinical	0.39	(0.21 - 0.71)	0.96	(0.47 - 1.96)	0.39	(0.21 - 0.70)
	Non-clinical	0.40	(0.21 - 0.76)	0.77	(0.36 - 1.63)	0.42	(0.22 - 0.79)

^a ^Extended Parallel Process Model

^b ^Percent of respondents included in EPPM category

^c ^Johns Hopkins Hospital (JHH)

^d ^MOR is the multinomial odds ratio provided in the multinomial logistic regression which compares the odds ratios between this category and the Low Threat/Low Efficacy category as the reference with respect to a particular characteristic category against its reference category, adjusted for key demographic characteristics: gender, age, hours/week worked, highest education level completed and children/marital status.

When questioned about potential modifiers of willingness to respond (conditional willingness to respond), WTR increased to 83.7% if a vaccine/daily preventive medications were made available compared to WTR of 72% if asked. Clarifications of potential worker safety issues considerably reduced WTR rates, compared to WTR if asked: if vaccine/daily preventive medications were *not *available or provided for all staff (55.4%); if personal protective equipment was *not *available for all staff (36.3%), if personal risk of quarantine existed (53.4%) and regardless of event severity (67.7%). WTR under each of these conditions was statistically significant different (p < 0.001 using McNemar's test) from WTR if asked.

## Discussion

Willingness to respond is a critical component of effective hospital readiness and sustainability in emergencies. Hospitals are expected to withstand considerable challenges during an influenza pandemic, including surge capacity, patient triage, infection control, delaying non-emergent surgical procedures, and expanding ICU capacities. Withstanding these challenges, especially during the pandemic peak, is an "all hands on deck" requisite. Hospital staff will be expected to work additional hours and do so under significant strain, including risk of one's safety and fear of potential transmission of illness to family members. Worker absenteeism is expected to be one of the most significant challenges for hospitals during the peak of an influenza pandemic (particularly during winter). Our findings further highlight the need to tackle the challenge of voluntary absenteeism in the context of healthcare organizational response capacity enhancement.

Over a quarter (28%) of the hospital staff surveyed indicated they are unlikely to respond to a pandemic if asked to report to duty. If the workers were required (and not just asked) to report to duty, the unwillingness to respond rate decreased to 18%. That, however, would leave nearly one of every six employees from a large urban tertiary care hospital not reporting for work - at a time they would be most needed in their respective work roles. When further probed if they would respond to a pandemic "regardless of severity", almost one third (32%) of surveyed staff indicated they are unlikely to do so.

These results are consistent with findings from a 2008 survey of healthcare workers at a large university hospital in Germany, where more than one third (36.2%) of respondents indicated that they would not come to work in the event of an influenza pandemic [21]. Our findings also expand upon previous research assessments of willingness to respond among other sectors of the healthcare community. The response willingness rates observed in the current study are higher, for example, than those from a 2005 survey performed in health departments, in which nearly half of surveyed local public health workers reported unwillingness to respond to an influenza pandemic [22]. The present study's willingness rates also exceed the rates of anticipated response willingness to pandemic influenza among prehospital emergency medical providers in Australia, where 43% of respondents indicated unwillingness to report in such an event [23]. However, the willingness rates in the current study are lower than those from a group of 2006-2007 surveys of public health workers in the US, where 86% were willing to respond even if only asked and not required to do so [11]. Our present study's willingness rates are also lower than the rates among nurses in Hong Kong, where 84% endorsed that they would be prepared to take care of patients infected by a potential avian influenza outbreak [7].

Of note, the present study's relatively low response willingness rates were observed in different types of employees. Unadjusted for key demographic factors, nurses were less likely to respond [OR(95%CI): 0.61 (0.45, 0.84)], compared to physicians. Indeed, previous smaller-scale survey-based research on hospital-based workers' willingness to respond to pandemic influenza identified nurses as less likely (44%) than physicians (74%) to indicate definitively their willingness to respond to an influenza pandemic scenario - a finding consonant with our study data [8].

When analyzing the statements most significantly associated with increased willingness to respond, several interesting insights become readily apparent. Perceived importance of one's role in the organizational response was strongly associated with response willingness. As this outcome is consistent with previous work [11,22], it becomes evident that a key step is to better educate health workers as to their designated roles during this unique type of emergency scenario, and then motivate them with an understanding of why this role makes a difference.

A new statement added to this survey proved to be the most strongly associated factor with willingness to respond if asked - whether one agrees to work more hours in performance of their duties during an emergency. A quarter of respondents indicated they would not agree to do so, and they were 14 times less likely to respond during a pandemic if asked than those who were willing to work more hours. Indeed, previous studies have suggested that willingness to work extra hours is an important issue, and varies among different types of disasters [24]. This rather straightforward question may thus serve as an excellent screening instrument to identify staff at risk for voluntary absenteeism during an event. Administrators could use this question in advance to further identify and suggest solutions to problems that may hinder workers from responding to unexpected events (i.e., promoting pre-event plans for dependents).

Another unique statement added to this survey proved to be strongly associated with willingness to respond if asked - whether one considers his or her peers likely to report. It is tempting to attribute this strong association to a cause-and-effect of what psychological models refer to as "subjective norm" [25], but when specifically probed if they will be more likely to respond if their peers will do so, the level of response willingness remained almost unchanged. A plausible interpretation would be that workers likely to respond feel their peers are likely to do the same, but the actual impact of the "subjective norm" on those not willing to respond is quite limited.

As in previous analyses of healthcare workers' willingness to respond, we address in this study the gaps in willingness to respond through systematic application of a behavioral model that addresses cognitive and emotional dynamics of response willingness attitudes. As a theoretical model built upon decades of prior research on fear campaigns and health risk messaging, the EPPM describes how an interaction of threat appraisals (perceived severity and likelihood) and efficacy appraisals (self and response efficacy) may influence behavioral responses to messages with fear content.

Using the EPPM, we can see how hospital workers' individual degrees of perceived threat ('concern') and perceived efficacy ('confidence') influence their willingness to respond. Indeed, consistent with results in public health workers [11], we have found that individuals who had a perception of high threat and high efficacy - i.e., those who fit a 'concerned and confident' profile in the EPPM framework - had a high rate of declared self-reported willingness to respond (if required) to pandemic flu, which was about nine times [OR(95%CI): 9.25 (5.94, 14.40)] higher than those fitting a 'low threat/low efficacy' EPPM profile. But unlike for public health workers [9], the threat ('concerned') aspect of preparedness for pandemic flu has shown no real impact on willingness to respond among hospital workers. In fact, the 'high threat/high efficacy' EPPM profile was not significantly different than the 'low threat/high efficacy' EPPM profile in their willingness to respond (both if asked and if required). One could thus argue that in the context of the current national emphasis on pandemic flu as a viable threat, the differences in recognition of the level of the threat should not receive great emphasis in hospital-based preparedness training.

In our study, staff with the 'high threat/high efficacy' EPPM profile were older, more likely to work long hours, have elder family members in their care, be a clinical staff member (but not a physician) and to work in the emergency department, when compared to staff in the 'low threat/low efficacy' profile.

The results of this survey point toward several additional practical strategies for reducing voluntary hospital worker absenteeism. While 72% of the respondents felt they could safely arrive to work, only 57% perceived their work environment as safe. The response willingness rate decreased to 36% if personal protective equipment were not made universally available to all employees, but increased to 84% if pharmaceutical countermeasures (pandemic vaccine and antiviral drugs) were secured for all workers. Indeed, this finding resonates with data from previous hospital-based research, which identified preferential access to antivirals and personal protective equipment (PPE) for employees and/or their families as among the most effective proposed absenteeism mitigation strategies in a pandemic influenza scenario [26]. Notifying workers of the measures taken to address health and safety concerns is of utmost importance in addressing pandemic challenges.

Certain limitations to the current study must be acknowledged. Most important is the fact that while we have strived to minimize social desirability bias in the construction and phrasing of the survey instrument content, any survey-based study is not fully predictive of actual behavior during an event. The representative sample included a study population of nearly 3,500 workers who participated in the survey, comprising about 18% of the JHH employees. Broad announcements from hospital leadership encouraging participation in the study were directed toward all employees encompassing the entire range of job descriptions.

Despite these caveats, ascertaining disposition of workers at a large, tertiary care hospital toward fulfilling pandemic flu response expectations nonetheless has value for local, state, and national readiness and response efforts and related training needs assessments.

## Conclusions

Our findings point to the gaps in willingness to respond within the hospital infrastructure, and the EPPM as a useful framework to assess these gaps and to identify remedies to some of the key issues. Our data indicate that for hospital workers, the level of confidence (perceived efficacy) in one's role during a pandemic is a particularly influential factor on willingness to respond in this scenario. Moreover, our findings reveal that several strategies - including promoting pre-event plans for dependents at home and ensuring the supply of personal protective equipment, vaccines and antiviral drugs for all hospital employees - may allow hospital leaders to design, implement, and evaluate risk communication messaging and training programs focused on emergency response willingness in their institutions.

## Competing interests

The authors declare that they have no competing interests.

## Authors' contributions

RDB conceived and designed the study; wrote the manuscript and coordinated its development; edited the manuscript; and operationalized the study's conceptual framework. DJB designed the study, co-wrote the manuscript and co-coordinated its development; edited the manuscript; and operationalized the study's conceptual framework. CBT co-wrote the manuscript; operationalized the study's conceptual framework; and conducted the statistical analyses. EBH edited the manuscript; and contributed to survey implementation. CLC edited the manuscript; and contributed to the manuscript framework and conceptual design. CMW contributed to survey implementation. NLS contributed to survey implementation. HSG contributed to survey implementation. JML reviewed and edited the manuscript; and contributed to the manuscript framework and conceptual design. All authors read and approved the final manuscript.

## Pre-publication history

The pre-publication history for this paper can be accessed here:

http://www.biomedcentral.com/1471-2458/10/436/prepub
